# Trying to cope with everyday life—Emotional support in municipal elderly care setting

**DOI:** 10.3402/qhw.v7i0.19613

**Published:** 2012-12-13

**Authors:** Margaretha Norell Pejner, Kristina Ziegert, Annica Kihlgren

**Affiliations:** 1 School of Health and Medical Sciences, Örebro University, Örebro, Sweden; 2 School of Social and Health Sciences, Halmstad University, Halmstad, Sweden

**Keywords:** Support, maintain strength, municipal care, the elderly

## Abstract

Emotional support is considered to be important to older patients because it is a contributing factor to experiencing good health and it has been shown that it can prevent depression after a hip fracture. Opinions differ on whether emotional support falls within the field of nursing, and studies also show that nurses in an elderly home care setting fail when it comes to giving emotional support. The aim of this study was to explore reasons for registered nurses to give emotional support to older patients in a municipal home care setting. The study was conducted using Grounded Theory. Data collection was carried out through interviews with 16 registered nurses. The inclusion criteria were emotional support given to patients aged 80 years and above living in ordinary or sheltered housing and who were in need of help from both the home help service and registered nurses. The results show that the main concern of emotional support was “Trying to relieve the patient from their emotions so they are able to cope with everyday life.” This core category illustrates how registered nurses tried to support the patients’ own strength, so that they were able to move forward. Registered nurses consider that they could support the patients because they give them access to, or could create access to, their emotions, but there were also times when they felt helplessness and as a result, consciously opted out. The results also indicate that registered nurses were keen to give emotional support. To develop patient-centered elderly care, more knowledge of emotional support and the elderly's need for this support is required.

Emotional support to older patients with health limitations is considered to be a factor to experience subjective health (Wang & Stumbo, 2009; White, Philogene, Fine, & Sinha, 2009). Besides increasing the feeling of subjective health, it has been shown that emotional support can reduce the risk of depression after a hip fracture (Shyu et al., 2009).

Some believe that emotional support must come from a family member or a person with whom the older patient has a relationship (de Jong Gierveld, van Groenou, Hoogerndoorn, & Smit, 2009; Okamoto & Harasawa, 2008). Lee (2010) maintains that older patients considered emotional support from professionals in connection with disease to be important, since it makes it easier for them to adapt to the situation caused by disease and aging. Chao (2012) also concludes that older patients are able to experience a higher level of life satisfaction when they receive emotional support from professionals.

Older people in Sweden who are in need of care have the right to apply for assistance from a handling officer to move to sheltered housing or to receive home help service in ordinary housing (SFS 2001: 453). Regardless of the older person's living situation, the usual organizational solution is that registered nurses (RNs) in a home care setting will be called from the home help service team in the event that the older person needs help (Nilsson, Lundgren, & Furåker, 2009). RNs describe their work situation as stressful, and say that it is hard to manage tasks and meetings with patients (Josefsson, Sonde, Winblad, & Robins Wallin, 2007). They know that they are able to influence their daily work situation by deciding which intervention should be initiated for the patient (Josefsson et al., 2007). One intervention often mentioned by RNs in relation to the care of the older patient is support (Manley, Webster, Hale, Hayes, & Minardi, 2008; Schein, Gagnon, Chan, Morin, & Grondines, 2005). In one study conducted in an elderly home care setting, it was found that the registered nurse (RN)-supportive interventions are defined as being directed toward both the patient and the environment (Norell, Ziegert, & Kihlgren, 2012). The same study also showed that in some situations where the patient expressed a need for emotional support, the RN could ignore the situation, despite the requirement to meet the patient's physical, social, mental, cultural and spiritual needs as a part of nurses’ competence (Socialstyrelsen, 2005).

In nursing, the term “emotional support” is described based on its context (Chalco et al., 2006; Law, 2009; Skilbeck & Payne, 2003). In the area of palliative care, emotional support is described as comforting interventions given in relation to the patient's disease with the purpose of facilitating the patient's feelings of anxiety (Law, 2009; Skilbeck & Payne, 2003). Chalco et al. (2006) connected emotional support with interventions such as information and education, so the patient was able to handle their situation and complete their treatments properly. Law (2009) argues that it is the nurse's knowledge about the disease and dying which helps the patient to process their emotions. Lindström and Eriksson (2006) also consider professionals to be important to help the patient in the process of addressing their emotions and situation to make sound choices, which can be seen as health promotion.

Skilbeck and Payne (2003) believe that it is difficult for nurses in a home care context to provide emotional support since this requires an interpersonal meeting, which is time consuming. Nurses often work under time pressure, leading to patients not receiving the emotional support that they require (Skilbeck & Payne, 2003). Law (2009) also argues that nurses in a home care context are unable to provide emotional support, despite their repeated contact with the patient, because they feel that they do not have the time or the knowledge required to cope with it. To develop elderly home care, it is important to find out which factors influence the RN so that they are able to engage with older patients when they express a need for emotional support in a home care context. The aim of this study was to explore reasons for registered nurses to give emotional support to elderly patients in a municipal elderly home care setting. We also aimed to gain a deeper understanding of how the process of emotional support is managed by registered nurses.

## Method

The study was conducted using Grounded Theory, which offers techniques for studying and responding to social realities (Corbin & Strauss, 2008). Corbin and Strauss’ (1990) version of Grounded Theory has its origins in Pragmatism and Symbolic Interactionism and provides the ability to see processes between actions/interactions that often occur between complex social phenomena. A process is seen as a continuous action in relation to a determinate purpose to reach a goal with a problem or a situation (Corbin & Strauss, 2008; Strauss & Corbin, 1998), and actors can choose actions to influence a course of events (Corbin & Strauss, 1990).

### Participants, procedure, and analysis

The study was performed in a municipality in the southwestern part of Sweden as part of a project focusing on RNs’ interventions with elderly patients in a municipal elderly care setting. The study population consists of RNs who are employed by the municipality or by a contractor working in liaison with the municipality. Whatever the mode of operation, the RN works on a consultative basis and is independent. They are responsible for the health care of the patient living in sheltered or ordinary housing.

Initially, contact was made with relevant directors who received information about the aims and procedures of the study. RNs were later informed about the study orally by their directors.

When all the RNs had received information, they were personally contacted by telephone and asked to participate in the study. The time and place of the meeting was decided by the RN who wanted to participate, and they received information once again both in writing and orally in connection with the data collection. The information consisted of the aims of the study and the procedure. The RNs gave their written consent to participate in the study.

Data were collected through interviews, which were recorded. The inclusion criteria were RNs’ experiences of emotional support given to elderly patients aged 80 years and above living in ordinary or sheltered housing, and who were in need of help from both the home help services and RNs.

Open sampling was initially used to be open to all possibilities and dimensions. To ensure openess, the first question in the intervew was a general question: “What is your experience of giving emotional support?” RNs were allowed to speak freely without interruptions. In the case of something being unclear, suppementary questions were asked afterward. The questions asked were: “Can you give an example? Can you elaborate on that?”

Every interview was transcribed immediately afterward, and analysis using open coding was started. The text was read several times and passages with similar content were compared, grouped together, and assigned a code. The codes were compared with the aim of finding connections or differences. During the analysis process, ideas and assumptions were documented using memos in the form of diagrams, figures, or recorded media. By returning to the memos and using them to compare and test new categories, they were used throughout the whole analysis procedure.

After the first interview and analysis, the next RN was contacted for new data collection and analysis. The first analysis and its memos were compared with the second. This procedure continued until data collection number six, when similar codes were grouped in preliminary categories.

In connection with data collection number seven, a theoretical sampling was used to explore the preliminary categories which proved to be significant cases. The reason why preliminary categories seemed significant was that the nurses’ stories were similar. Statements and situations described by the RN were repeated. To avoid misunderstandings between the RN and the researcher, the preliminary categories were tested by addressing the category by asking the nurse questions. In this data collection, the first interview question was the same as in previous cases, but the following questions were asked to confirm or eliminate preliminary categories. The questions asked were: “Does it have to be like that? Why did you do that?” On those occasions when some new fact was revealed, the RN was encouraged to tell us about it.

During data collection and previous analysis, axial coding was also carried out.

Questions asked in the preliminary categories were: “What happened?,” “Why?,” “What did the RN do?,” and “What was the purpose of this action?” The objective of the questions was to increase the level of the content by bringing the text together. Preliminary categories became categories, and sub-categories were formed, which, in turn, were tested against each other. The meaning of the core category was detected at an early stage but was difficult to define. When the core category was finally established, it was tested against all the other categories in a process moving from the core category through all categories to and fro to establish it or to reevaluate the hierarchy. All categories were also tested against the original interview to test the relevance of the category. The review revealed that two categories coincided, and so they were combined. To finally establish the categories, all of the authors carried out a joint review of the material and the analyses and the categories were tested against the study participants.

A total of 16 out of 29 RNs employed were interviewed. The interviews lasted between 40 and 90 min. Their professional experience was between 5 and 32 years; 11 of them had a specialist nurse education as district nurses and one was specialized in geriatrics. One of the district nurses had also had training in organization and leadership, and four had had training in palliative care.

### Ethics

This study is not considered to pose a risk to the participants. Personal data such as names or identification numbers allowing an individual to be traced have not been used. The study was carried out in accordance with the ethical requirements of consent, voluntariness, and confidentiality (SFS 2003: 460). The study was approved by the regional research ethics committee (Reg. no. 2009/456).

## Findings

The findings showed that the reason why RNs give emotional support to a group of patients aged 80 years and above in an elderly home care setting involved “Trying to relieve the patient from their emotions so they are able to cope with everyday life” (Table I), which the core category illustrates. In the categories, it appears that RNs considered that they could manage patients’ emotions because “The patients give me access to their emotions” and because “I can create access to patient emotions”, but there were also situations in which the RNs “Feel helplessness and consciously drop out”, which led to emotional support not being provided.

**Table I:** Trying to relieve the patient from their emotions so they are able to cope with everyday life.

The patient gives me access to their emotions	I can create access to patients’ emotions	Feeling helplessness and consciously drop out
Because of my profession	By my approach	I lack the tools
Because I am an outsider	By trigging emotions	I feel like a pawn

### Trying to relieve the patient from their emotions so they are able to cope with everyday life

The RNs express how patients’ memories from the past, actual life situation, disease, or an incident could affect the patient in such a way that they found it difficult to manage their situation. Reasons for this included different kinds of emotions causing the patient to become stuck in a state of mind that tired them out and, in turn, impeded their ability to deal with the event. The RNs give words to how they try to support patients’ ability or wishes to move forward in their situation.

(RN3) She is almost 90 years and a very powerful lady who really is very sick. It's about having the power, to mediate power, to support the power she has. When you are 90 years it is clear that life is approaching its end, but this doesn't mean you want to die.

The RNs found it difficult to be expressive of the emotional support they provided, and on several occasions it was noted that their actions was committed without reflection. Emotional support was not considered to be a specific nursing activity but could be included in nursing since it was an act of compassion. The RNs also found that emotional support was important and told how they alternated between having the ability and trying to create the opportunity to support patients in their emotions.

### The patient gives me access to their emotions

The RNs considered that their position within the organization contributed to them having access to patients’ emotions. As one RN expressed it:

(RN 13) I have the key to the lock.

The reasons why they considered that they have access were “Because of my profession” and “Because I am an outsider”.

### Because of my profession

The RNs considered emotional support to be part of their professional skill of knowing when a patient is in need of emotional support.

(RN5) You can go to the home of the same patient many times and the conversations are quite different depending on the patient's mood, daily condition and how they feel. Sensing this is part of our profession.

Other than considering emotional support to be part of their duty of assisting the patient, they also recognized that their profession meant that the patient confided in them. Confidence existed for two reasons. First, being an RN involves having a position in the organizational hierarchy. The elderly regarded the RN with respect and as a member of the community in which they could confide. Second, the patient knew that RN had a duty of confidentiality.

### Because I am an outsider

Being an RN in such an organization means not being a person who is involved in patients’ everyday lives or involved in situations in which the patients do not feel at a disadvantage, such as hygiene, going to the toilet, or receiving help with eating. It is about respect for the patients.

(RN 7) I don't help the patient use the toilet or the shower while we are talking about their emotions.

Another aspect of being an outsider was that the patients prefer to speak about their emotions with someone that they do not risk hurting or coming into conflict with.

(RN 13) It is easier to talk with me than to talk to their children or spouse, to have a neutral person to talk to.

### I can create access to patients’ emotions

Sometimes the patients could have difficulties talking about their emotions for various reasons or were not ready to express what they felt at that moment. The RNs tried to adapt according to the situation and were prepared to come back at another time to find out what was weighing the patient down.

(RN 11) Sometimes you have to invent things in order to come back and try to keep in touch.

It appeared that the RNs used two different strategies to reach patients’ emotions: “By my approach” and “By trigging emotions.”

### By my approach

The RNs considered that the way they approach the patient was important, since this could influence how the patient responded and, depending on the situation, the RN could act in different ways. One way was by letting the patient talk about the things that worried them. The RNs believed that the patient could find relief in this and recover the strength they needed to move forward, conscious of the fact that, most of the time, they could not do anything about the situation except listen. The important thing was to let the patient talk about it and show the patient that they were open to hearing the patient's emotions, which was achieved by showing the patient that they were both mentally and physically present.

(RN 3) A lot of emotional support is about being present, that you're there for them; it's very important that they know that I'm available.

### By trigging emotions

Another way of creating access to patients’ emotions was through provoking those emotions that the RN thought the patient was experiencing. This was done whether it involved positive emotions that could be strengthened with happiness or if something was weighing the patient down. The reason for provoking emotions was that the RN believed the patient would feel better if they could talk about it.

(RN 4) And by talking he puts world on it himself.

### Feeling helplessness and consciously drop out

There were occasions when the RNs consciously chose to turn away because they felt unable to influence the situation.

(RN 5) One must be completely open when you arrive at the patient's home and during the first few minutes you should use your sense of what the conversation should be about, what my support should be or if I should leave immediately.

Reasons why the RNs chose to opt out were found to be principally due to “I lack the tools” and “I feel like a pawn.”

### I lack the tools

The most common reason why RNs chose to interrupt a conversation dealing with patients’ emotions was because of time constraints. The RNs did not have enough time to sit down to listen. Opting out of providing emotional support also related to the fact that they knew it was better not to do it than to be stressed doing it.

(RN 7) Some days there's no use even thinking about it, because you know, you do not have the time.

Another reason for RNs to opt out was because they realized that they did not always see or understand what it was about, or know how to deal with it.

(RN 7) Sometimes you feel like a chameleon. You are constantly trying to do the right thing. However, it is not always the case that I judge the situation correctly, since I do not always understand what is going on.

### I feel like a pawn

In some homes, the situation could be upsetting because of disagreements between family members or their spouses. The RNs felt that they were used to taking a stand for or against different positions in situations in which they did not want to be involved in, and decided to opt out.

(RN 6) She refused to accept his partner and did not even look at him. He became desperate and sought the support of every person who got there. It didn't matter if it was the district nurse, the home care provider or the newspaper boy, he only sought support, but we could not give it.

## Discussion

The core category “Trying to relieve the patient from their emotions so they are able to cope with everyday life” illustrates how the RNs try to support the patients’ own strength and desire to gain the willpower to be able to continue. Supporting the patients’ own strength can be seen as a health promotion approach. Sullivan (1989) argues that nurses are particularly suited to dealing with patients’ emotions because they know about bridging patients’ health limitations and thus help them to structure their lives. Lindström and Eriksson (2006) add that this requires a health promotion approach.

Reasons why the RNs considered themselves suited to dealing with patients’ emotions include: *“*The patient gives me access to their emotions” or “I can create access to patients’ emotions”, which the categories indicate. The RNs considered giving emotional support as part of their profession, which both Sullivan (1989) and Law (2009) also believe, but remarkably the RNs also believe that the organizational solution contributes to their ability to give emotional support, which the subcategory “I am an outsider” illustrates. Both Skilbeck and Payne (2003) and Law (2009) contradict this. They believe that nurses in a home care setting organization do not have the time required or the knowledge to give emotional support, something that is partially seen in this study and reflected in the subcategory “I lack the tools.”

It is noteworthy that the RNs seems to be keen to support patients’ emotions and try to find solutions to adapt to the patients’ situation, which is seen in the category *“*I can create access to patients’ emotions.” Adapting to the patients’ situation to provide an interaction can be seen as patient-centered care (Hobbs, 2009; Robinson, Callister, Berry, & Dearing, 2008). Robinson et al. (2008) elucidate three components required to achieve patient-centered care: communication, shared decision-making, and support for self-management. It seems that by making use of the strategies “By my approach” and “By trigging emotions,” RNs are trying to achieve one or more of the three components. The first of these is communication. Here, one can see that the RNs are trying to adapt to the patients’ situation, either by showing their presence or by provoking emotions when the patients have difficulty expressing them. The second, shared decision-making includes taking the patients’ perspective (Robinson et al., 2008). Berwick (2009) talks about individualization and customization, and concludes that to achieve this there needs to be flexible systems in place to be able to meet patient needs. Josefsson et al. (2007) showed in their study, which was conducted in a home care setting, that the nurses themselves had the opportunity to influence the individual care situation and had their superiors’ support to do so. This indicates that this form of care organization has a certain flexibility. It is reasonable to believe that the RNs in this study take advantage of the flexibility that exists in the organization to meet the individual needs of the elderly. The third, support for self-management, is—according to Robinson et al. (2008)—combined with follow-up visits. In the category “I can create access to patients’ emotions,” it was shown that the RNs do not drop the subject but leave it so that they could come back to find out what the patient's emotions were about.

Patient-centered care often takes the perspective of the individual impairment to strengthen the patient's own ability to manage their illness (Coleman, Parry, Chalmers, & Min, 2006; Hobbs, 2009; Silver, Keefer, & Rosenfeld, 2011). Lezwijn et al. (2011) argue that in the care of elderly patients, one cannot only focus on the patient's limited health conditions but should also ascertain which resources are needed to strengthen the patient. Perhaps this is what the RNs are trying to do. However, to identify these resources, more knowledge about the importance of emotional support for the health of the elderly patient is required.

## Method discussion

Using Grounded Theory is appropriate, as the purpose of this study was to explore reasons and how the process of emotional support is managed by RNs. Although Grounded Theory provides the opportunity to explore ongoing processes (Corbin & Strauss, 1990, 2008; Strauss & Corbin, 1998), there have been difficulties in elucidating them on the grounds that there have been various parallel processes. This has led to difficulties arranging the categories and subcategories. Using interviews as the data collection method has allowed RNs to provide clarification on the occasions where there has been ambiguity. Another thing that has helped to clarify the results has been returning the categories in the study to the participants to see if they recognize themselves. Patton (2002) considers that this is a way of increasing reliance in the material. Unfortunately, the study consists of a limited number of participants, which can be considered a weakness.

## Conclusion

The main reason why RNs give emotional support to a group of elderly patients in a municipal care setting is that they are trying to relieve the patient of their emotions so that they are able to cope with everyday life. This core category shows that RNs not only focus on the patients’ limited health but also try to stimulate the patients’ own resources. This is done because RNs believe that the patients give them access or that they are able to create access to the patient’ emotions. Occasionally, it becomes apparent that RNs refuse to provide emotional support because they feel that they do not have the required tools or are concerned that they would potentially get involved in something that they cannot control. To develop patient-centered elderly care, more knowledge is needed of the elderly patients’ views on the need for emotional support and how it affects the health of the elderly.

## References

[CIT0001] Berwick D (2009). What patient-centered should mean: Confessions of an extremist. Health Affairs.

[CIT0002] Chalco K, Wu D. Y, Mestanza L, Munoz M, Llaro K, Guerra D (2006). Nurses as providers of emotional support to patients with MDR–TB. International Nursing Review.

[CIT0003] Chao S-F (2012). Functional disability and psychological well-being in later life. Does source of support matter?. Aging & Mental Health.

[CIT0004] Coleman E, Parry C, Chalmers S, Min S-J (2006). The care transitions intervention. Results of a randomized controlled trial. Archives of Internal Medicine.

[CIT0005] Corbin J, Strauss A (1990). Grounded Theory research: Procedures, canons and evaluative criteria. Qualitative Sociology.

[CIT0006] Corbin J, Strauss A (2008). Basics of qualitative research.

[CIT0007] de Jong Gierveld J, van Groneou M, Hoogendoorn A, Smit J (2009). Quality of marriages in later life and emotional and social loneliness. Journal of Gerontology: Social Sciences.

[CIT0008] Hobbs J (2009). A dimensional analysis of patient-centered care. Nursing Research.

[CIT0009] Josefsson K, Sonde L, Winblad B, Wallin Robins T-B (2007). Work situation of registered nurses in municipal elderly care in Sweden: A questionnaire survey. International Journal of Nursing Studies.

[CIT0010] Law R (2009). Bridging worlds’: Meeting the emotional needs of dying patients. Journal of Advanced Nursing.

[CIT0011] Lee G. E (2012). Predictors of adjustment to nursing home life of elderly residents: A cross-sectional survey. International Journal of Nursing Studies.

[CIT0012] Lezwijn J, Vaandrager L, Naaldenberg J, Wagwmakers A, Koelen M, van Woerkum C (2001). Healthy ageing in a salutogenic way: Building the HP 2.0 framework. Health and Social Care in the Community.

[CIT0013] Lindström B, Eriksson M (2006). Contextualizing salutogenesis and Antonovsky in public health development. Health Promotion International.

[CIT0014] Manley K, Webster J, Hale N, Hayes N, Minardi H (2008). Leadership role of consultant nurses working with older people: A co-operative inquiry. Journal of Nursing Management.

[CIT0015] Nilsson K, Lundgren S, Furåker C (2009). Registered nurse's everyday activities in municipal health care: A study of diaries. International Journal of Nursing Practice.

[CIT0016] Norell M, Ziegert K, Kihlgren A (2012). Dealing with daily emotions—supportive activities for the elderly in a municipal care setting. International Journal of Qualitative Studies on Health and Wellbeing.

[CIT0017] Okamoto K, Harasawa Y (2008). Emotional support from family members and subjective health in caregivers of the frail elderly at home in Japan. Archives of Gerontology and Geriatrics.

[CIT0018] Patton M. Q (2002). Qualitative research & evaluation methods.

[CIT0019] Robinson J, Callister L, Berry J, Dearing K (2008). Patient-centered care and adherence: Definitions and applications to improve outcomes. Journal of the American Academy of Nurse Practitioners.

[CIT0020] Schein C, Gagnon A, Chan L, Morin I, Grondines J (2005). The Association between specific nurse case management interventions and elder health. Journal of American Geriatrics Society.

[CIT0021] Shyu Y-I, Cheng H-S, Teng H-C, Chen M-C, Wu C-C, Tsai W-C (2009). Older people with hip fracture: Depression in the postoperative first year. Journal of Advanced Nursing.

[CIT0022] Silver G, Keefer J, Rosenfeld P (2011). Assisting patients to age in place: An innovative pilot program utilizing the patient centered care model (PCCM) in home care. Home Health Care Management & Practice.

[CIT0023] Skilbeck J, Payne S (2003). Emotional support and the role of clinical nurse specialists in palliative care. Journal of Advanced Nursing.

[CIT0024] Socialstyrelsen (2005). Kompetensbeskrivning för legitimerad sjuksköterska.

[CIT0025] Strauss A, Corbin J (1998). Basics of qualitative research techniques and procedures for developing grounded theory.

[CIT0026] Sullivan G (1989). Evaluating Antonovsky's salutogenic model for its adaptability to nursing. Journal of Advanced Nursing.

[CIT0027] Svensk författningssamling (SFS 2001: 453) Socialtjänstlag.

[CIT0028] Svensk författningssamling (SFS 2003: 460) Lag om etikprövning av forskning som avser människor.

[CIT0029] Wang Y, Stumbo N. J (2009). Factors affecting quality of life for community-dwelling older adults with a disability: Implications for therapeutic recreation practice and research. Annual in Therapeutic Recreation.

[CIT0030] White A. N, Philogene S, Fine L, Sinha S (2009). Social support and self-reported health status of older adults in the United States. American Journal of Public Health.

